# Genome‐wide association studies on resistance to powdery mildew in cultivated emmer wheat

**DOI:** 10.1002/tpg2.20493

**Published:** 2024-07-28

**Authors:** Dhondup Lhamo, Genqiao Li, George Song, Xuehui Li, Taner Z. Sen, Yong‐Qiang Gu, Xiangyang Xu, Steven S. Xu

**Affiliations:** ^1^ USDA‐ARS, Crop Improvement and Genetics Research Unit, Western Regional Research Center Albany California USA; ^2^ USDA‐ARS Peanut and Small Grains Research Unit Stillwater Oklahoma USA; ^3^ Department of Plant Sciences North Dakota State University Fargo North Dakota USA

## Abstract

Powdery mildew, caused by the fungal pathogen *Blumeria graminis* (DC.) E. O. Speer f. sp. *tritici* Em. Marchal (*Bgt*), is a constant threat to global wheat (*Triticum aestivum* L.) production. Although ∼100 powdery mildew (*Pm*) resistance genes and alleles have been identified in wheat and its relatives, more is needed to minimize *Bgt*’s fast evolving virulence. In tetraploid wheat (*Triticum turgidum* L.), wild emmer wheat [*T. turgidum* ssp. *dicoccoides* (Körn. ex Asch. & Graebn.) Thell.] accessions from Israel have contributed many *Pm* resistance genes. However, the diverse genetic reservoirs of cultivated emmer wheat [*T. turgidum* ssp. *dicoccum* (Schrank ex Schübl.) Thell.] have not been fully exploited. In the present study, we evaluated a diverse panel of 174 cultivated emmer accessions for their reaction to *Bgt* isolate *OKS(14)‐B‐3‐1* and found that 66% of accessions, particularly those of Ethiopian (30.5%) and Indian (6.3%) origins, exhibited high resistance. To determine the genetic basis of *Bgt* resistance in the panel, genome‐wide association studies were performed using 46,383 single nucleotide polymorphisms (SNPs) from genotype‐by‐sequencing and 4331 SNPs from the 9K SNP Infinium array. Twenty‐five significant SNP markers were identified to be associated with *Bgt* resistance, of which 21 SNPs are likely novel loci, whereas four possibly represent emmer derived *Pm4a*, *Pm5a*, *PmG16*, and *Pm64*. Most novel loci exhibited minor effects, whereas three novel loci on chromosome arms 2AS, 3BS, and 5AL had major effect on the phenotypic variance. This study demonstrates cultivated emmer as a rich source of powdery mildew resistance, and the resistant accessions and novel loci found herein can be utilized in wheat breeding programs to enhance *Bgt* resistance in wheat.

## Core Ideas

- A large portion (66%) in a panel of 174 cultivated emmer accessions was highly resistant to powdery mildew.
- Genetic basis of *Blumeria graminis* f. sp. *tritici* (*Bgt*) resistance in the panel was analyzed using genome‐wide association studies (GWAS) based on 50,174 genotype‐by‐sequencing (GBS) and 9K single nucleotide polymorphisms (SNPs).
- Twenty‐five significant SNP markers were identified, with four SNPs representing known emmer‐derived *Pm* resistance genes.
- Twenty‐one novel SNP markers were predominantly found on chromosomes 1B, 2A, 2B, and 7A.

**Abbreviations**
*Bgt*
: *Blumeria graminis* f. sp. *tritici*
Blink: Bayesian‐information and linkage‐disequilibrium iteratively nested keyway
Chr: chromosome
FarmCPU: Fixed and random model Circulating Probability Unification
GBS: genotype‐by‐sequencing
GWAS: genome‐wide association studies
IT: infection type
LD: linkage disequilibrium
LOESS: locally estimated scatterplot smoothing
*Pm*
: powdery mildew
PVE: phenotypic variance explained
Q‐Q plot: quantile‐quantile plot
SNP: single nucleotide polymorphism
WT: Welch's *t*‐test

## 1 INTRODUCTION

Powdery mildew is a foliar disease caused by the highly adaptable fungal pathogen *Blumeria graminis* (DC.) E. O. Speer f. sp. *tritici* Em. Marchal (*Bgt*), which emerged during wheat (*Triticum aestivum* L., 2*n* = 6*x* = 42, AABBDD) domestication in Fertile Crescent ∼10,500 years ago and has since spread throughout the world along with its host (Sotiropoulos et al., 2022). Powdery mildew poses an important threat to global wheat production due to the rapid evolution and diversification of *Bgt* via mutation and selection (Menardo et al., 2016). Powdery mildew causes an estimated yield loss of 7.6%–19.9% annually in wheat (Bowen, 1991; Conner et al., 2003), and its occurrence is expected to rise with changing climates and agricultural practices (Kloppe et al., 2022; Tang et al., 2017).

Host resistance is considered the most effective and sustainable approach to manage powdery mildew. So far, 99 permanently designated powdery mildew resistance (*Pm*) genes at 63 distinct loci (*Pm1* to *Pm69*, *Pm18* = *Pm1c*, *Pm22 *= *Pm1e*, *Pm23 *= *Pm4c*, *Pm31 *= *Pm21*, *Pm48 *= *Pm46*, and allelic *Pm8*/*17*) have been reported, among which 43 are allelic (Xu et al., 2023). Thirteen *Pm* resistance genes have been cloned, of which *Pm1a*, *Pm2a*, *Pm3b/8/17*, *Pm5e*, *Pm12/21*, *Pm41*, *Pm60/MIWE18/MIIW172*, and *Pm69/PmG3M* encode nucleotide‐binding leucine‐rich repeat proteins; *Pm4*, *Pm24*, and *WTK* encode protein kinases; *Pm38/Lr34/Yr18/Sr57* encodes ABC transporter; and *Pm46*/*Lr67*/*Yr46*/*Sr55* encodes hexose transporter (Y. Li et al., 2023). Although many *Pm* resistance genes have been identified, more are needed to negate *Bgt*’s fast evolving pathogenicity. In addition, a considerable number of the known *Pm* genes are derived from wild relatives of wheat and require pre‐breeding to eliminate linkage drag that compromises the overall crop performance (Summers & Brown, 2013). Therefore, a continuous search for novel *Pm* genes is of agronomic importance for developing cultivars with robust and durable resistance.

Tetraploid wild emmer wheat [*T*. *turgidum* ssp. *dicoccoides* (Körn. ex Asch. & Graebn.) Thell., 2*n *= 4*x *= 28, AABB], the AABB genome donor of common wheat and the progenitor of durum wheat [*T. turgidum* ssp. *durum* (Desf.) Husn., 2*n *= 4*x *= 28, AABB], represents a large reservoir of genetic variation for beneficial traits (L. Huang et al., 2016; Klymiuk et al., 2019). Wild emmer wheat has contributed many *Pm* resistance genes to modern wheat cultivars (Table 1), of which only *Pm41*, *Pm69/PmG3M*, and *TdPm60* have been cloned. Given that most wild emmer accessions deposited in global germplasm banks remain untapped, the wild emmer gene pool is still considered an important resource for powdery mildew resistance (Klymiuk et al., 2019).

**TABLE 1:** Powdery mildew resistance genes (*Pm*) originating from tetraploid emmer wheat.

*Pm* gene	Chr	Resistance donor	Reference
*Pm3k*	1AS	*T. turgidum* ssp. *dicoccoides*	Yahiaoui et al., 2006
*Pm4a*	2AL	*T. turgidum* ssp. *dicoccum*	Ma et al., 2004
*Pm50*	2AL	*T. turgidum* ssp. *dicoccum*	Mohler et al., 2013
*MIIW70*	2BS	*T. turgidum* ssp. *dicoccoides*	Z. Liu et al., 2012
*Pm26*	2BS	*T. turgidum* ssp. *dicoccoides*	Rong et al., 2000
*Pm42*	2BS	*T. turgidum* ssp. *dicoccoides*	Hua et al., 2009
*Pm49/MI5323*	2BS	*T. turgidum* ssp. *dicoccum*	Piarulli et al., 2012
*MIAB10*	2BL	*T. turgidum* ssp. *dicoccoides*	Maxwell et al., 2010
*MIZec1*	2BL	*T. turgidum* ssp. *dicoccoides*	Mohler et al., 2005
*Pm64*	2BL	*T. turgidum* ssp. *dicoccoides*	D. Zhang et al., 2019
*Pm41*	3BL	*T. turgidum* ssp. *dicoccoides*	G. Li et al., 2009; M. Li et al., 2020
*Pm16*	5BS	*T. turgidum* ssp. *dicoccoides*	Chen et al., 2005
*Pm30*	5BS	*T. turgidum* ssp. *dicoccoides*	Z. Liu et al., 2002
*MI3D232*	5BL	*T. turgidum* ssp. *dicoccoides*	H. Zhang et al., 2010
*Pm36*	5BL	*T. turgidum* ssp. *dicoccoides*	Blanco et al., 2008
*MIRE*	6AL	*T. turgidum* ssp. *dicoccum*	Chantret et al., 2000
*Pm31*	6AL	*T. turgidum* ssp. *dicoccoides*	C. Xie, Sun, Ni, et al., 2003; C. Xie et al., 2004
*Pm69/PmG3M*	6BL	*T. turgidum* ssp. *dicoccoides*	Y. Li et al., 2023; Wei et al., 2020; W. Xie et al., 2012
*MIIW72*	7AL	*T. turgidum* ssp. *dicoccoides*	Ji et al., 2008
*PmG16*	7AL	*T. turgidum* ssp. *dicoccoides*	Ben‐David et al., 2010
*TdPm60*	7AL	*T. turgidum* ssp. *dicoccoides*	Y. Li et al., 2021; Q. Wu et al., 2022
*Pm5a*	7BL	*T. turgidum* ssp. *dicoccum*	Law & Wolfe, 1966

Abbreviation: Chr, chromosome.

Cultivated emmer wheat [*T. turgidum* ssp. *dicoccum* (Schrank ex Schübler) Thell., 2*n *= 4*x* = 28, AABB] is domesticated from wild emmer about 10,000 years ago (Willcox, 1998). It has evolved substantially with hybridization, selection, and enrichment of naturally occurring mutations (Badaeva et al., 2015). Like its wild progenitor, cultivated emmer also harbors a valuable genetic resource to improve resistance to biotic and abiotic stresses (Sharma et al., 2019; Zaharieva et al., 2010). Five *Pm* resistance genes have been identified from cultivated emmer lines using biparental mapping populations (Table 1). The enriched genetic reservoirs present in cultivated emmer germplasm have not been fully exploited. In this study, we took advantage of a diverse panel of 174 cultivated emmer accessions to identify new sources of powdery mildew resistance and reveal novel resistance loci for powdery mildew using genome‐wide association studies (GWAS).

## 2 MATERIALS AND METHODS

### 2.1 Plant materials and genotyping

A panel of 174 cultivated emmer accessions provided by the USDA‐ARS National Small Grains Collection (NSGC, Aberdeen, ID) was used to identify powdery mildew resistance loci in this study. These emmer accessions originated from 32 countries and were previously genotyped using the 9K SNP Infinium iSelect array and genotype‐by‐sequencing (GBS)‐derived single nucleotide polymorphism (SNP) markers which were filtered as described in Lhamo et al. (2023).

### 2.2 Evaluating responses of the emmer panel to *Bgt* isolate *OKS(14)‐B‐3‐1*

The emmer panel was evaluated for responses to *Bgt* isolate *OKS(14)‐B‐3‐1* (Table S1) as described (Tan et al., 2018; Xu et al., 2023). *OKS(14)‐B‐3‐1*, which was collected, characterized, and maintained by USDA‐ARS Plant Science Research Unit at Raleigh, North Carolina, is a representative *Bgt* isolate of the Great Plains. *OKS(14)‐B‐3‐1* is virulent to *Pm2*, *Pm3g*, *Pm7*, *Pm8*, and *Pm9* but avirulent to *Pm1*, *Pm2*, *Pm3a*, *Pm3b*, *Pm3c*, *Pm3d*, *Pm3e*, *Pm4a*, *Pm4b*, *Pm5a*, *Pm5b*, *Pm5d*, *Pm6*, *Pm12*, *pm13*, *Pm16*, *Pm17*, *Pm20*, *Pm21*, *Pm25*, *Pm34*, *Pm35*, *Pm37, Pm59, Pm63*, and *Pm65* (G. Li, Xu, et al., 2019; G. Li, Cowger, et al., 2019; Tan et al., 2018, 2019). The moderate resistance of *OKS(14)‐B‐3‐1* makes it ideal for gene discovery via GWAS because it allows expression of most *Pm* resistance genes.

Briefly, the experiment was conducted in a randomized complete block design with two replicates. In each replicate, 10 plants from each accession were grown and tested. The *Bgt* isolate *OKS(14)‐B‐3‐1* was increased on wheat cultivar Jagalene, and plants were inoculated at the two‐leaf stage and grown under natural light at 20 ± 2°C in a greenhouse at the USDA‐ARS Peanut and Small Grains Research Unit (Stillwater, Oklahoma, USA). Plant phenotyping was performed after 10 days of inoculation, and the infection type (IT) of each tested plant was scored using the 0–4 scale (Z. L. Wang et al., 2005) in which no visible symptom was scored as IT = 0, observation of necrotic flecks as IT = 0;, small colonies with few conidia as IT = 1, colonies with moderately developed hyphae but few conidia as IT = 2, colonies with generously developed hyphae and abundant conidia but no coalescing as IT = 3, and coalescing colonies with both hyphae and conidia as IT = 4 (Tan et al., 2018). Plants were classified as highly resistant (IT = 0, 0; and 1), moderately resistant (IT = 2), moderately susceptible (IT = 3), and highly susceptible (IT = 4). Durum wheat line KL‐B (Gill et al., 2021) derived from “Khapli” cultivated emmer (Briggle, 1966) was used as the resistant check, and a durum genetic stock Rusty (Klindworth et al., 2006) was used as the susceptible check.

**Core Ideas**

- A large portion (66%) in a panel of 174 cultivated emmer accessions was highly resistant to powdery mildew.
- Genetic basis of *Blumeria graminis* f. sp. *tritici* (*Bgt*) resistance in the panel was analyzed using genome‐wide association studies (GWAS) based on 50,174 genotype‐by‐sequencing (GBS) and 9K single nucleotide polymorphisms (SNPs).
- Twenty‐five significant SNP markers were identified, with four SNPs representing known emmer‐derived *Pm* resistance genes.
- Twenty‐one novel SNP markers were predominantly found on chromosomes 1B, 2A, 2B, and 7A.

### 2.3 Statistical analysis

Disease responses of the emmer panel were tested for normality using the Shapiro–Wilk test in R (Shapiro & Wilk, 1965). Because the distribution was not normal (*p *< 0.05; Table S2), the nonparametric Fligner–Killeen test was performed in R to evaluate homogeneity between replicates (Conover et al., 1981). The mean of homogenized experiments (*p *> 0.05) was calculated and used for association mapping.

### 2.4 Marker–trait association and linkage disequilibrium analyses

GWAS was conducted to identify SNP markers associated with powdery mildew resistance. Five statistical models were tested for association mapping in GAPIT3 (J. Wang & Zhang, 2021), and the best fit model was chosen based on the quantile‐quantile (Q‐Q) plot. Fixed and random model Circulating Probability Unification (FarmCPU; X. Liu et al., 2016) was implemented for the 9K SNP array, and Bayesian‐information and linkage‐disequilibrium iteratively nested keyway (Blink; M. Huang et al., 2019) for the GBS dataset. Manhattan plots were generated using the “CMplot” R package (https://github.com/YinLiLin/CMplot; Yin et al., 2021). Considering a *p*‐value threshold of 0.05, a Bonferroni‐corrected ‐log_10_(*p*) of 5 and 6 was applied for the 9K SNP array and GBS, respectively. To narrow down the SNPs that show a significant effect (*p* ≤ 0.05) on the phenotypic variation between the alternative alleles, Welch's *t*‐test was performed (Lu & Yuan, 2010). Gene annotations for significant SNPs were assigned based on the closest gene identified near the physical position of SNPs using the reference genomes of hexaploid wheat Chinese Spring RefSeq v1.0 (IWGSC et al., 2018) and RefSeq v2.1 (Zhu et al., 2021) and wild emmer accession Zavitan WEWSeq v2.0 (Zhu et al., 2019; Table S3).

Linkage disequilibrium (LD) was calculated by measuring the squared allele frequency correlation (*r* ^2^) between pairs of SNPs in TASSEL 5 (Bradbury et al., 2007), with a sliding window of 25 markers for GBS and full matrix for the 9K SNP array based on the SNP density. The locally estimated scatterplot smoothing (LOESS) was used to fit LD decay curve in R (Cleveland et al., 2017). The LD decay distance or point was estimated based on the physical distance at which the *r* ^2^ dropped to half of its maximum value, where *r* ^2^ = 1 implies a complete LD and *r* ^2^ = 0 implies an absence of LD.

## 3 RESULTS

### 3.1 Disease responses of the emmer panel

**FIGURE 1:**
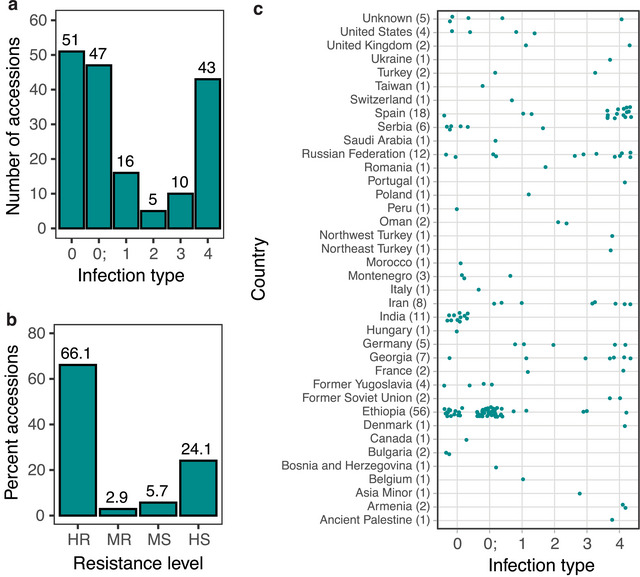
Disease response of the cultivated emmer wheat panel to *B. graminis* f. sp. *tritici* isolate *OKS(14)‐B‐3‐1*. (a) Infection type (IT), (b) resistance level of emmer accessions, and (c) relationship between emmer's country of origins and IT responses. Each dot represents individual emmer accession from the panel. Accessions with IT scores of 0, 0; and 1 are considered highly resistant (HR), 2 as moderately resistant (MR), 3 as moderately susceptible (MS), and 4 as highly susceptible (HS). The total number of emmer accessions from each country of origin is represented in bracket (*n*).

The emmer panel consisting of 174 accessions was evaluated for disease response to *Bgt* isolate *OKS(14)‐B‐3‐1*. The infection types of all emmer accessions in the panel are listed in Table S1. A bimodal distribution was observed in the emmer panel, with a group of 98 accessions displaying high resistance (IT = 0, 0;) and another group of 43 accessions displaying high susceptibility (IT = 4; Figure 1a). A total of 51 cultivated emmer accessions (29.3%) exhibited immune response to *Bgt* (IT = 0). Intermediate IT responses were found at low frequencies. Overall, 66.1% of cultivated emmer accessions were considered highly resistant (IT = 0, 0; and 1), 2.9% were moderately resistant (IT = 2), 5.7% were moderately susceptible (IT = 3), and 24.1% were highly susceptible (IT = 4; Figure 1b). The major portion of highly resistant emmer accessions originated from Ethiopia (53 out of 56) and India (11 out of 11), among which 91% of Ethiopian (51) and 100% of Indian (11) accessions exhibited very low IT score of 0; and/or 0 (Figure 1c, Table 2). In addition, several regions representing a smaller portion of the emmer panel show high resistance including Serbia (five out of six accessions), former Yugoslavia (four out of four), and the United States (four out of four). However, variation in susceptibility was observed in other European and Asian countries. Highly susceptible emmer accessions originated mainly from Spain (15 out of 18), Georgia (four out of eight), and Russian Federation (5 out of 12).

**TABLE 2:** Summary of cultivated emmer accessions resistant to *B. graminis* f. sp. *tritici* isolate *OKS(14)‐B‐3‐1*.

Infection type (IT)	Resistance level^a^	Origin or source^b^	No. of accession
0, 0;	HR	Bosnia and Herzegovina	1
		Bulgaria	2
		Canada	1
		Ethiopia	51
		Former Yugoslavia	4
		Georgia	1
		Hungary	1
		India	11
		Iran	2
		Italy	1
		Montenegro	2
		Morocco	1
		Peru	1
		Russian Federation	4
		Saudi Arabia	1
		Serbia	5
		Spain	1
		Taiwan, China	1
		Turkey	1
		United States	2
		Unknown	4
1	HR	Georgia	1
		Belgium	1
		Ethiopia	2
		France	1
		Germany	2
		Iran	1
		Montenegro	1
		Poland	1
		Spain	2
		Switzerland	1
		United Kingdom	1
		United States	2
2	MR	Oman	2
		Germany	1
		Romania	1
		Serbia	1

^a^ IT scores of “0”, “0;”, and “1” are considered highly resistant (HR), “2” as moderately resistant (MR).

^b^ Origin or source information was based on U.S. National Plant Germplasm System (https://npgsweb.ars‐grin.gov/gringlobal).

### 3.2 Genome‐wide associations

**FIGURE 2:**
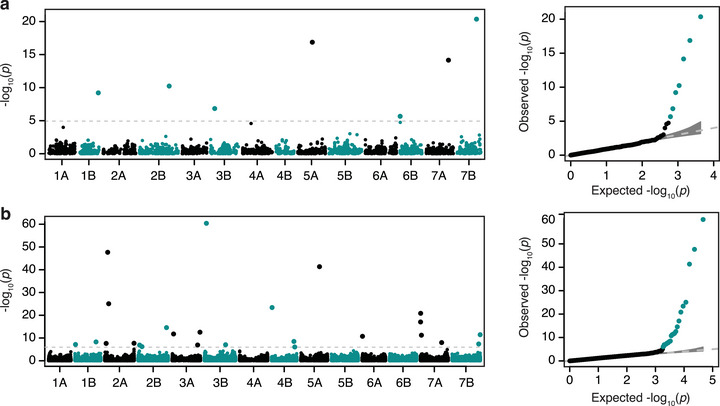
Manhattan plots displaying single nucleotide polymorphisms (SNPs) from (a) the 9K SNP array and (b) genotype‐by‐sequencing (GBS) of the emmer panel that are associated with powdery mildew resistance. Quantile‐quantile (Q‐Q) plots are shown on the right. A Bonferroni‐corrected ‐log_10_(*p*) value was set as the significance threshold (gray dashed line). Dots represent SNP markers for marker‐trait association.

To identify genetic loci contributing to *Bgt* resistance in the emmer panel, GWAS was performed using two different genotypic datasets, which consist of 4331 SNPs from the 9K SNP array (Figure 2a) and 46,383 SNPs from GBS (Figure 2b). Five statistical models were primarily tested for marker–trait associations. Based on the resulting Q‐Q plots, FarmCPU was found to be the best fit model for the 9K SNP array (Figure 2a) and Blink for the GBS dataset (Figure 2b). The stringent Bonferroni‐corrected thresholds were applied to reduce false positive marker–trait associations. This resulted in seven SNPs from the 9K SNP array that were significantly [‐log_10_(*p*) ≥ 5] associated with powdery mildew resistance, which contributed 1.8%–23.9% of the phenotypic variance explained (PVE; Figure 2a). These SNP markers were located on chromosomes 1B, 2B, 3B, 5A, 6B, 7A, and 7B. Using the GBS dataset, 25 SNPs were identified to significantly [‐log_10_(*p*) ≥ 6] associate with powdery mildew resistance, which explained 0.3%–27% of the phenotypic variance (Figure 2b). These SNP markers were present on all chromosomes except for 1A, 4A, 5B, and 6B.

### 3.3 LD decay analyses

**FIGURE 3:**
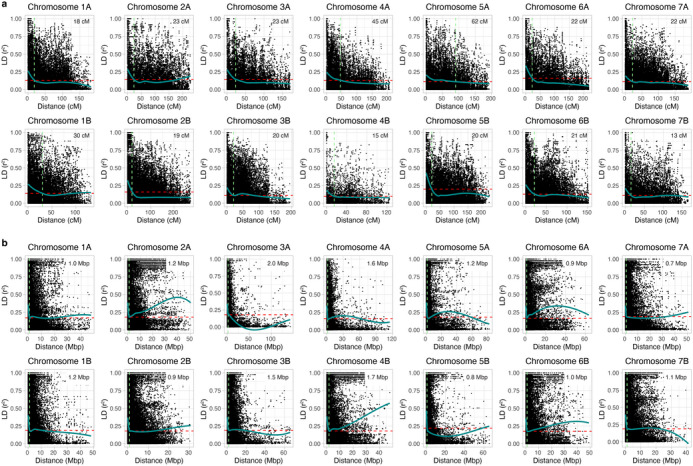
Scatter plots displaying the chromosome‐wise linkage disequilibrium (LD) decay using (a) the 9K single nucleotide polymorphism (SNP) markers and (b) the genotype‐by‐sequencing (GBS) markers of the cultivated emmer panel. LD decay is represented by the squared allele correlation coefficient (*r* ^2^) between pairwise SNPs over a physical distance (cM or Mbp). The dark‐green line represents LD decay curve fitted using the LOESS model. A horizontal red dashed line is the LD threshold, set at half of its maximum *r* ^2^ value. A vertical light‐green dashed line indicates the physical distance at which LD between markers falls below the LD threshold, with the LD decay point or distance presented at the top right corner of each plot.

LD decay was performed on all chromosomes to estimate the genetic distance required for significant SNP markers to be considered in linkage or present at the same trait locus (Figure 3). The genetic distance at which the LD dropped to half of its maximum value (*r* ^2 ^= 0.10–0.20) using the 9K SNP markers varied broadly, with chromosomes 4B and 7B at 13–15 cM; chromosomes 1B, 4A, and 5A at 30–62 cM; and the remaining chromosomes at 18–23 cM (Figure 3a). Five 9K SNP markers on chromosomes 1B, 2B, 3B, 5A, and 7B were found in close proximities to some of the GBS markers, with the genetic distance between close markers ranging between 32 and 128 Mbp (Table 3). Assuming a rough conversion of 1 cM as 1 Mbp, the genetic distance of SNP markers on 5A (32 Mbp) only was possibly within the estimated LD decay point of 62 cM (Table 3, Figure 3a). Therefore, S5A_495771738 and wsnp_Ku_c51039_56457361 could potentially share the same locus for powdery mildew resistance, whereas the other SNPs likely represent unique resistance loci.

**TABLE 3:** Significant single nucleotide polymorphisms (SNPs) of the cultivated emmer panel associated with powdery mildew resistance.

SNP marker	Chr	Position (Mbp)	Marker code	Marker position (cM)	‐log_10_(*p*)	PVE (%)	Alleles	WT	R allele	Close *Pm* genes
S1B_18564537	1B	18.56			7	0.6	A/G	*	A	
S1B_561151612	1B	561.15			8	1.7	A/C	***	A	
wsnp_Ex_c955_1827567	1B	688.77	IWA4935	136.89	9	3.3	A/G	***	G	
S2A_38557560	2A	38.56			8	0.7	C/T	*	C	
S2A_78559456	2A	78.56			48	27.0	C/G	**	G	
S2A_105044730	2A	105.04			25	2.8	T/C	**	T	
S2A_775441196	2A	775.44			8	0.9	G/A	*	A	*Pm4a*
S2B_42277651	2B	42.28			7	0.5	T/A	**	T	
S2B_104577357	2B	104.58			6	0.7	T/C	**	T	
wsnp_Ex_c24135_33382318	2B	715.03	IWA2872	208.46	10	7.4	G/A	**	A	*Pm64*
S2B_754153049	2B	754.15			15	1.8	A/C	***	A	
S3A_38433125	3A	38.43			12	1.9	G/A	***	G	
S3A_675295616	3A	675.3			7	0.8	A/T	***	A	
wsnp_Ra_c16264_24873670	3B	17.77	IWA7647	30.13	7	2.1	C/T	***	C	
S3B_55404315	3B	55.4			60	17.1	T/C	***	C	
S4B_593136973	4B	593.14			8	0.6	T/C	***	T	
S4B_615781324	4B	615.78			6	0.3	A/C	***	A	
S5A_495771738	5A	495.77			41	12.9	A/G	***	A	
wsnp_Ku_c51039_56457361	5A	527.92	IWA7135	102.41	17	21.2	T/C	***	T	
wsnp_Ex_c1143_2196102	6B	8.41	IWA1495	0	6	1.8	G/A	**	G	
S7A_6298660	7A	6.3			21	1.0	G/C	***	G	
S7A_26878648	7A	26.88			11	1.3	T/G	***	G	
wsnp_Ex_c61603_61581218	7A	700.7	IWA4437	159.52	14	8.9	A/G	***	A	*PmG16*
wsnp_Ex_c6961_11997446	7B	708.12	IWA4593	136.21	20	23.9	T/G	***	T	*Pm5a*
S7B_743147375	7B	743.15			11	4.8	C/T	***	C	

*Note*: SNP markers starting with “S” are derived from GBS, and “wsnp” from the 9K SNP array.

Abbreviations: Chr, chromosome; *Pm*, powdery mildew; PVE, phenotypic variance explained; QTL, quantitative‐trait locus; R allele, resistant allele; SNP, single nucleotide polymorphism; WT, Welch's *t*‐test.

*p ≤ 0.05. **p ≤ 0.01. ***p ≤ 0.001.

For the GBS markers, the LD dropped to half (*r* ^2^ = 0.16–0.22) for all chromosomes at ≤ 2 Mbp, with the rapid LD decay point at 0.7–0.9 Mbp for chromosomes 2B, 5B, 6A, and 7A and the gradual LD decay point at 1.7–2.0 Mbp for chromosomes 3A and 4B (Figure 3b). Several significant GBS markers were found in close proximities to each other on chromosomes 2A, 2B, 4B, and 7A; however, the genetic distance between those close markers ranged between 26 and 40 Mbp for chromosome 2A, 62 Mbp for 2B, 23 Mbp for 4B, and 21 Mbp for 7A (Table 3). Because the distance between close GBS SNP markers exceeded the LD decay point (≤ 2 Mbp), they were considered different genomic loci.

### 3.4 SNPs with significant allelic effect and their resistance sources

To assess SNPs that show significant differences in disease responses between the alternative alleles, Welch's *t‐*test was performed. In total, 25 SNPs from both genotypic datasets exhibited significant allelic effect (*p *< 0.05), which were largely present on chromosomes 1B, 2A, 2B, and 7A (Table 3). Eighteen SNPs showed minor effect (< 5% PVE), two with moderate effect (5%–10% PVE) and five with major effect (>10% PVE). Among the five major effect SNP markers, two were from the 9K SNP array and three from GBS. The 9K SNP markers such as wsnp_Ku_c51039_56457361 on 5A and wsnp_Ex_c6961_11997446 on 7B explained 21.2% and 23.9% of the phenotypic variance, respectively (Table 3). The GBS markers such as S2A_78559456, S3B_55404315, and S5A_495771738 explained 27%, 17.1%, and 12.9% of the phenotypic variance, respectively (Table 3).

**FIGURE 4:**
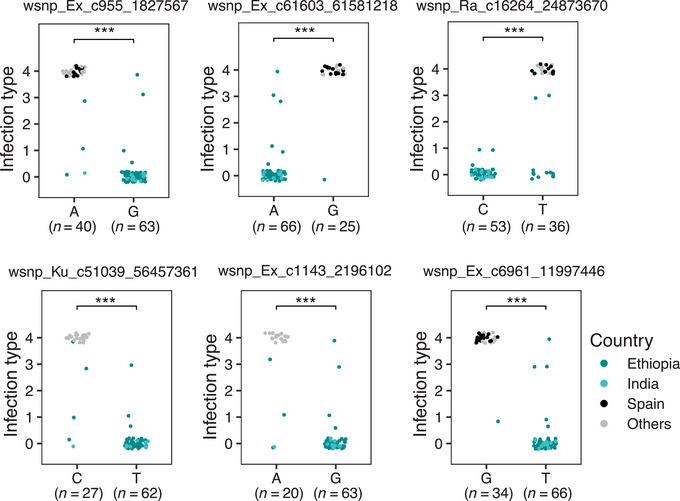
Alternative alleles of the significant 9K single nucleotide polymorphism (SNP) markers with resistance originating from emmer accessions of Ethiopia and India. Resistant alleles of all accessions from the resistant sources were compared with susceptible alleles from the highly susceptible (IT = 4) sources. Dots represent accessions with the alternative alleles, and (*n*) represents the number of accessions with a resistant allele or a susceptible allele. The significance levels of *** corresponds to *p* < 0.0001.

**FIGURE 5:**
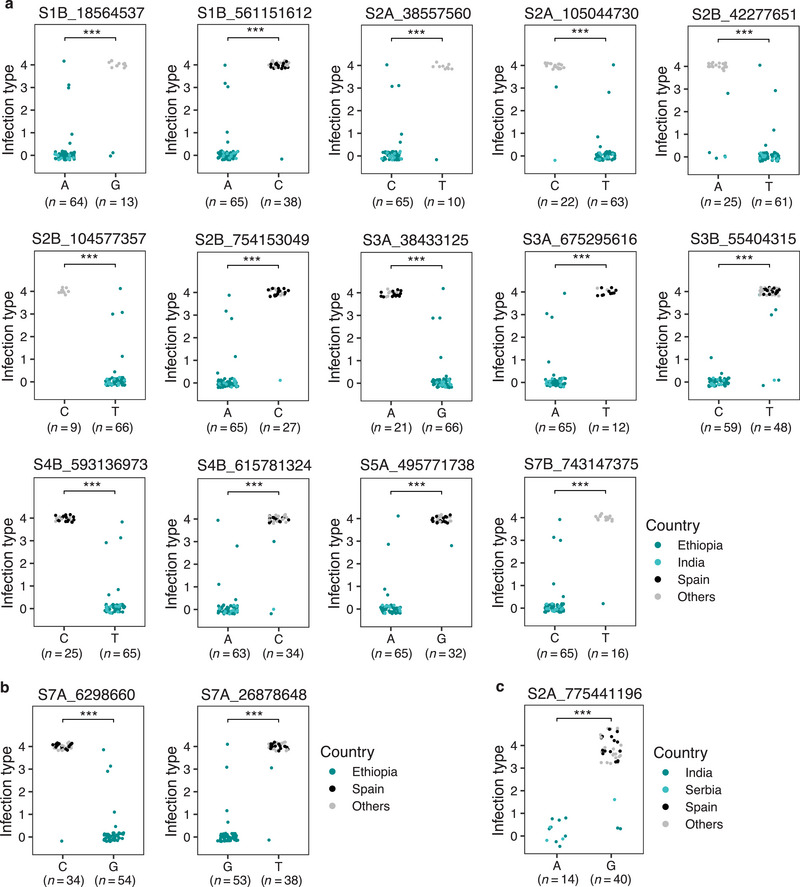
Alternative alleles of the significant genotype‐by‐sequencing (GBS) single nucleotide polymorphism (SNP) markers with resistance originating from emmer accessions of (a) Ethiopia and India, (b) Ethiopia only, and (c) India and Serbia. Resistant alleles of all accessions from the resistant sources were compared with susceptible alleles from the highly susceptible (IT = 4) sources. (*n*) represents the number of accessions with a resistant allele or a susceptible allele. The significance levels of *** corresponds to *p* < 0.0001.

To determine the sources of resistance, we sorted the alternative alleles of significant SNPs for all emmer accessions from the 9K SNP array (Table S4) and GBS (Table S5). Because the majority of the resistant cultivated emmer accessions originated from Ethiopia and India, their contributions to resistant alleles were evaluated in comparison to highly susceptible accessions (IT = 4) from Spain and other countries (e.g., Georgia, Russian Federation, and Iran) for the significant 9K SNP markers (Figure 4) and the GBS markers (Figure 5). The resistant alleles of all the 9K SNP markers except for wsnp_Ex_c24135_33382318 originated from emmer accessions of Ethiopia and India, whereas the susceptible alleles of four SNP markers excluding wsnp_Ku_c51039_56457361 and wsnp_Ex_c1143_2196102 were contributed by emmer accessions of Spain along with other countries (Figure 4, Table S4).

For the GBS dataset, the resistant alleles of 14 significant SNPs were contributed by Ethiopian and Indian emmer accessions (Figure 5a), two SNPs on chromosome 7A by Ethiopia only (Figure 5b), and one SNP on 2A by Indian and Serbian emmer accessions (Figure 5c). Resistant accessions of former Yugoslavia, Montenegro, the United States, and few from Russian Federation have also contributed resistant alleles for some SNP markers (Tables S4 and S5). The susceptible alleles of 11 significant SNPs were contributed by the accessions from Spain along with other countries (Figure 5a–c).

## 4 DISCUSSION

Powdery mildew pathogen *Bgt* poses a serious threat to wheat‐growing regions due to its rapidly changing virulence, resulting in ineffectiveness of deployed *Pm* resistance genes in wheat cultivars within a short time span (Müller et al., 2022). G. Li, Xu, et al. (2019) and Summers and Brown (2013) previously evaluated the response of 1292 wheat landraces and historical cultivars to three *Bgt* isolates and found 80%–90% accessions were highly susceptible to *OKS(14)‐C‐2‐1*, *OKS(14)‐B‐3‐1*, and *Bgt15*, suggesting the sources of resistance to these *Bgt* isolates are rare in hexaploid wheat germplasm. *OKS(14)‐B‐3‐1* was identified as the least virulent among the three isolates although it is virulent to differential lines carrying *Pm2*, *Pm3g*, *Pm7*, *Pm8*, and *Pm9*. Because more highly resistant accessions (4%) were identified for *OKS(14)‐B‐3‐1* than the other two isolates, this allowed the detection of three resistance loci on chromosome 2BL, while not a single resistance locus was detected for the highly virulent *OKS(14)‐C‐2‐1* due to the limited number of highly resistant accessions (1.3%) found in the hexaploid germplasm (G. Li, Xu, et al., 2019). Therefore, the present study utilized *Bgt* isolate *OKS(14)‐B‐3‐1* of Great Plains for disease evaluation to maximize the detection of powdery mildew resistance loci.

Tetraploid wild emmer and cultivated emmer wheat serve as rich sources of powdery mildew resistance and have contributed more than 25 *Pm* resistance genes (L. Huang et al., 2016; Klymiuk et al., 2019; Sharma et al., 2019; Zaharieva et al., 2010). However, almost all these *Pm* genes were derived from resistant accessions of Israeli origin, which were mapped using biparental populations (G. Li et al., 2009, 2021; Maxwell et al., 2010; Q. Wu et al., 2022; C. J. Xie, Sun, & Yang, 2003; D. Zhang et al., 2019). This suggests that a limited genetic diversity of emmer wheat has been explored in finding *Pm* resistance genes, and more remain yet to be identified.

To broaden powdery mildew resistance sources, in this study, we evaluated a diverse panel of 174 cultivated emmer accessions for reactions to *Bgt* isolate *OKS(14)‐B‐3‐1* and found 66% accessions to be highly resistant. Interestingly, most of the cultivated emmer accessions originating from Ethiopia (95%) and India (100%) are highly resistant, which make up 37% of the highly resistant accessions in the panel. So far, wild emmer accessions of Israeli origin have served as the major contributor of *Pm* resistance genes, while enriched resistance in Ethiopian and Indian cultivated emmer accessions remains unexploited except for “Khapli”, Indian cultivated emmer that provided powdery mildew and stem rust resistance (Reed, 1916; Williams & Gough, 1965). The resistant emmer accessions found in this study could serve as new resistance donors for modern durum and bread wheat cultivars.

To understand the genetic basis of *Bgt* resistance in the emmer panel, we performed association mapping and identified 25 SNP markers to be associated with powdery mildew resistance. More significant SNPs were identified using the GBS dataset (18) than the SNP array (7) because the SNP density was 10–20 times higher in the GBS dataset across 14 chromosomes of the emmer panel (Lhamo et al., 2023). In addition, the significant SNPs from the 9K SNP array were detected mostly on the BB genome (5) compared to the AA genome (2), whereas an equal number of significant SNPs were present on the AABB genomes using the GBS dataset (Table 3). This could possibly be caused by a lower marker density and/or an uneven marker distribution on AA genome compared to BB genome using the 9K SNP array. For instance, we detected four significant SNPs on the short and long arms of chromosome 2A using the GBS dataset, whereas none were found using the 9K SNP array (Table 3), probably because a large portion of the 9K SNP markers were distributed around the centromeric region of 2A (Cavanagh et al., 2013). Such factors may challenge the detection of significant SNPs with overlaps between the genotypic datasets.

To differentiate novel loci, the genomic regions of significant SNPs were compared with the previously reported *Pm* resistance genes in wild and cultivated emmer wheat. Four significant SNP markers identified in this cultivated emmer panel may be linked to the known *Pm* resistance genes of emmer wheat such as *Pm4a*, *Pm5a, Pm64*, and *PmG16*. For instance, the minor‐effect S2A_775441196 located at 775 Mbp was found near the genomic region of *BCD1231* (762 Mbp) marker linked to *Pm4a* at a distance of 4.8 cM (Ma et al., 2004). This GBS SNP marker was found closer to the flanking markers *Xics13* and *Xics43* (773–780 Mbp) of *Pm4b*, an allele of *Pm4a* (P. Wu et al., 2018). However, *Pm4b* is derived from Persian wheat [*T. turgidum* ssp. *carthlicum* (Nevski) Á. Löve & D. Löve] and *Pm4a* from Khapli cultivated emmer (Briggle, 1966). Analysis of resistant allele of the SNP marker (S2A_775441196) revealed its resistance sources to be contributed largely by Indian and Serbian accessions (Figure 5c); thus, it is more likely to represent *Pm4a*. The identification of a closer linked marker for *Pm4a* might be needed to resolve the positional discrepancy.

The 9K SNP marker wsnp_Ex_c24135_33382318 at 715 Mbp on chromosome 2BL shared overlapping regions with the SSR markers *Xwmc175* and *Xwmc332* (670–739 Mbp) associated with *Pm64* of wild emmer (D. Zhang et al., 2019). Thus, this genomic locus may represent the resistance provided by *Pm64*. However, the resistant allele of wsnp_Ex_c24135_33382318 is detected in only few accessions from Russian Federation, Iran, and Serbia (Table S4), suggesting the maintenance of *Pm64* in few cultivated emmer accessions post‐domestication from wild emmer. Another SNP marker is detected on 2BL at 754 Mbp (S2B_754153049) from the GBS dataset, providing a stronger allelic effect with resistance originating from cultivated emmer accessions of Ethiopia and India (Figure 5a). Other *Pm* resistance genes reported on 2BL of wild emmer include *MIZec1* (*Xwmc356* at ∼797 Mbp; Mohler et al., 2005) and *MIAB10* (*Xwmc445* at ∼799 Mbp; Maxwell et al., 2010). Whether SNP markers identified on 2BL of the cultivated emmer represent wild emmer‐derived *Pm* genes such as *Pm64*, *MIAB10*, or *MIZec1* requires further confirmations.

On chromosome 3B, only *Pm41* was reported on the long arm (3BL) of wild emmer accession IW2, and its linked marker *BE489472* was found at ∼763 Mbp (G. Li et al., 2009; M. Li et al., 2020). However, the two SNP markers (wsnp_Ra_c16264_24873670 and S3B_55404315) were found on the short arm 3BS and likely represent novel loci, which share Ethiopia and India as common origins for the resistant alleles (Figures 4 and 5a). These two SNP markers were considered distinct genomic loci based on LD decay analyses (Figure 3). Of the two, S3B_55404315 displays a major effect on the phenotype variance (Table 3).

The moderate effect wsnp_Ex_c61603_61581218 on chromosome 7AL shared overlapping region (∼701 Mbp) with *PmG16‐*associated markers *XstsBE445653* and *Xwmc525* (634–713 Mbp); therefore, these may represent the same locus (Ben‐David et al., 2010). *PmG16* is considered an ortholog of *Pm60/MlWE18*, which is positioned at around 724–726 Mbp based on the associated markers and collinearity (Y. Li et al., 2021; Q. Wu et al., 2022, 2021). Besides *Pm60*, *Pm69* is another cloned gene from wild emmer that is present in close proximity (∼716 Mbp; Y. Li et al., 2023; Wei et al., 2020; W. Xie et al., 2012). Two minor‐effect SNP markers were found on the short arm of 7A, which has not been previously reported in emmer wheat. In addition, the resistance source of these markers was specifically derived from Ethiopian accessions (Figure 5b), which further supports its novelty. Overall, chromosomes 2B and 7A of emmer wheat appear to be enriched with *Pm* resistance genes.

*Pm5a* is the only powdery mildew resistance gene found on chromosome arm 7BL of cultivated emmer “Yaroslav” (Law & Wolfe, 1966), and it is recently found to be located at ∼706 Mbp based on its sequence deposited in GenBank (J. Xie et al., 2020). Of the two SNP markers found on chromosome 7BL, the major effect wsnp_Ex_c6961_11997446 is present nearby *Pm5a*, with the distance of about 1.3 Mb, and potentially represents the same locus. Its resistant allele was present in 100% of highly resistant accessions (IT = 0) and the susceptible allele in 77% of highly susceptible accessions (IT = 4; Table S4), supporting a strong marker–trait association. Because *OKS(14)‐B‐3‐1* is avirulent to *Pm4a* and *Pm5a*, we were able to detect these cultivated‐emmer derived genes, explaining the reason behind the selected *Bgt* isolate and the robustness of our GWAS analyses.

The remaining 21 SNPs present on chromosomes 1B, 2A, 2B, 3A, 3B, 4B, 5A, 6B, and 7A are potentially novel genomic loci as *Pm* resistance genes were undetected on those regions in emmer wheat in earlier studies (L. Huang et al., 2016; Klymiuk et al., 2019; Sharma et al., 2019; Zaharieva et al., 2010). These SNP markers mostly displayed minor effects on the phenotypic variance although the majority exhibited strong allelic effects, signifying their importance in disease resistance. In addition, three novel loci on chromosomal arms 2AS, 3BS, and 5AL exhibited major effects and their sources of resistance derived from Ethiopian and Indian accessions except for S2A_78559456, with resistance originating from Serbia, Former Yugoslavia, and Montenegro (Table S5). The two SNP markers on 5AL (S5A_495771738 and wsnp_Ku_c51039_56457361) may represent a single locus based on the slow LD decay distance of chromosome 5A using the 9K SNP markers (Figure 3a). These major loci of cultivated emmer potentially contain new *Pm* resistance genes that can be introduced into elite wheat cultivars to maximize the genetic variability for powdery mildew resistance.

GWAS analyses serve as a starting point for discovering new powdery mildew resistance genes. Further validation of genomic loci identified in this study using biparental mapping population may uncover novel genes and provide genomic tools for cultivar development. For example, GWAS identified a resistance locus (*QPm.stars‐2BL3*) for powdery mildew resistance at 715.9 Mbp on chromosome 2B for the *Bgt* isolate *OKS(14)‐B‐3‐1* (G. Li, Xu, et al., 2019). Based on this result, Tan et al. (2019) developed a mapping population from a cross between PI 628024 × CItr 11349 and identified a new powdery mildew resistance gene *Pm63* in the terminal region of 2BL (710.3−723.4 Mbp). Notably, the same *Bgt* isolate *OKS(14)‐B‐3‐1* of Great Plains is used in this study, which is less virulent than those from the eastern United States (Cowger et al., 2018). Therefore, priority should be given to those trait loci conferring resistance to *Bgt* isolates from the eastern United States such as the Great Lake and Mid‐Atlantic regions.

In summary, through the present study, we identified cultivated emmer accessions from Ethiopia and India as rich yet unexplored sources of powdery mildew resistance. The identification of four loci corresponding to *Pm4a*, *Pm5a, Pm64*, and *PmG16* of emmer wheat signifies the strength of GWAS in predicting marker–trait associations for *Bgt* resistance. We additionally found three novel loci with major effects, and a fourth one associated with *Pm5a*. Resistant alleles of most novel loci were derived from resistant emmer accessions of Ethiopian and Indian origins. Therefore, the resistant accessions and loci found in this study represent new valuable resources for breeders to develop wheat cultivars with more durable resistance to powdery mildew. The results from disease evaluation and GWAS analysis presented herein will be useful for selecting parents in developing specific mapping populations to be used for linkage mapping of novel *Pm* genes.

## AUTHOR CONTRIBUTIONS

**Dhondup Lhamo**: Data curation; formal analysis; investigation; methodology; software; validation; visualization; writing—original draft; writing—review and editing. **Genqiao Li**: Data curation; investigation; methodology; validation; visualization; writing—review and editing. **George Song**: Data curation; writing—review and editing. **Xuehui Li**: Data curation; investigation; software; validation; visualization; writing—review and editing. **Taner Z. Sen**: Resources; validation; visualization; writing—review and editing. **Yong Gu**: Conceptualization; data curation; formal analysis; funding acquisition; investigation; methodology; project administration; resources; software; supervision; writing—review and editing. **Xiangyang Xu**: Conceptualization; data curation; funding acquisition; investigation; methodology; project administration; supervision; validation; visualization; writing—original draft; writing—review and editing. **Steven Xu**: Conceptualization; data curation; formal analysis; funding acquisition; investigation; methodology; project administration; resources; software; supervision; validation; visualization; writing—original draft; writing—review and editing.

## CONFLICT OF INTEREST STATEMENT

The authors declare no conflicts of interest.

## Supporting information


**Supplemental Table S1**. Responses of 174 cultivated emmer wheat accessions to isolate *OKS(14)‐B‐3‐1* of powdery mildew pathogen *B. graminis* f. sp. *tritici* (*Bgt*).

**Supplemental Table S2**. Normality and homogeneity tests of the replicated samples in response to *B. graminis* f. sp. *tritici* (*Bgt*) isolate *OKS(14)‐B‐3‐1*.

**Supplemental Table S3**. Physical locations of significant SNPs of the cultivated emmer panel on Chinese Spring and wild emmer reference genomes.

**Supplemental Table S4**. Alternative alleles of significant SNPs derived from the 9K SNP array for all cultivated emmer accessions.

**Supplemental Table S5**. Alternative alleles of significant SNPs derived from GBS for all cultivated emmer accessions.

## Data Availability

The accession numbers, sources, population and subpopulation clusters, and genotypic datasets of the cultivated emmer wheat panel used in this study were published in Lhamo et al. (2023) (Supplementary file 2). All other data supporting the findings of this study are present within the article and within supplementary materials published online. Table S1 contains disease evaluation data for all 174 cultivated emmer accessions. Table S2 provides results from normality and homogeneity tests of the replicated samples. Table S3 lists detailed chromosomal location and gene annotation of SNPs that are significantly associated with powdery mildew resistance in the cultivated emmer panel. Tables S4 and S5 show alternative alleles of significant SNPs for all cultivated emmer accessions.
