# Supplementary material for: Genome‐wide association studies on resistance to powdery mildew in cultivated emmer wheat
Source: Plant Genome. 2024 Jul 28;18(1):e20493. doi: 10.1002/tpg2.20493 (PMC11733656; doi:10.1002/tpg2.20493)
Supplement: Supplementary file 2 [file TPG2-18-e20493-s002.docx]

| **Supplemental Table S3.** Physical locations of significant SNPs of the cultivated emmer panel on Chinese Spring and wild emmer reference genomes. | | | | | | | | | | | |
| --- | --- | --- | --- | --- | --- | --- | --- | --- | --- | --- | --- |
| **Genotyping method** | **SNP** |  | **Chinese Spring IWGSC RefSeq v1.0** | | |  | **Chinese Spring IWGSC RefSeq v2.1** | |  | **Wild emmer acc. Zavitan WEWSeq v2.0** | |
|  |  | **Chr** | **SNP Position (Mbp)** | **Probe** | **Probe position (Mbp)** |  | **Probe** | **Probe position (Mbp)** |  | **Probe** | **Probe position (Mbp)** |
| 9K SNP array | wsnp_Ex_c955_1827567 | 1B | 688.77 | TraesCS1B01G794000LC | 688.76 |  | TraesCS1B03G1274600LC | 694.75 |  | TRIDC1BG071740 | 692.33 |
|  | wsnp_Ex_c24135_33382318 | 2B | 715.03 | TraesCS2B01G520500 | 715.03 |  | TraesCS2B03G1306200 | 723.47 |  | TRIDC2BG075120 | 729.22 |
|  | wsnp_Ra_c16264_24873670 | 3B | 17.77 | TraesCS3B01G042600LC | 17.77 |  | TraesCS3B03G0083400LC | 23.29 |  | TRIDC3BG005250 | 23.01 |
|  | wsnp_Ku_c51039_56457361 | 5A | 527.92 | TraesCS5A01G317000 | 527.92 |  | TraesCS5A03G0769300 | 528.61 |  | TRIDC5AG046940 | 532.61 |
|  | wsnp_Ex_c1143_2196102 | 6B | 8.41 | TraesCS6B01G013500 | 8.41 |  | TraesCS6B03G0029300 | 10.25 |  | TRIDC6BG001540 | 8.47 |
|  | wsnp_Ex_c61603_61581218 | 7A | 700.7 | TraesCS7A01G514800 | 700.69 |  | TraesCS7A03G1250700 | 705.84 |  | TRIDC7AG072320 | 711.36 |
|  | wsnp_Ex_c6961_11997446 | 7B | 708.12 | TraesCS7B01G443500 | 708.11 |  | TraesCS7B03G1197700 | 717.80 |  | TRIDC7BG069960 | 726.31 |
| GBS | S1B_18564537 | 1B | 18.56 | TraesCS1B02G038900 | 18.57 |  | TraesCS1B03G0078300 | 19.39 |  | TRIDC1BG004190 | 22.90 |
|  | S1B_561151612 | 1B | 561.15 | TraesCS1B01G576100LC | 561.16 |  | TraesCS1B02G576100LC | 567.82 |  | TRIDC1BG054160 | 580.42 |
|  | S2A_105044730 | 2A | 105.04 | TraesCS2A01G158100 | 105.03 |  | TraesCS2A03G0324700 | 109.70 |  | TRIDC2AG019780 | 113.96 |
|  | S2A_38557560 | 2A | 38.56 | TraesCS2A01G077200LC | 38.55 |  | TraesCS2A03G0164100LC | 43.13 |  | TRIDC2AG076920 | 762.81 |
|  | S2A_775441196 | 2A | 775.44 | TraesCS2A01G583700 | 775.44 |  | TraesCS2A03G1308400 | 771.50 |  | TRIDC2AG079130 | 773.14 |
|  | S2A_78559456 | 2A | 78.56 | TraesCS2A01G130800 | 78.56 |  | TraesCS2A03G0267700 | 83.26 |  | TRIDC2AG016070 | 87.03 |
|  | S2B_104577357 | 2B | 104.58 | TraesCS2B01G137600 | 104.57 |  | TraesCS2B03G0332400 | 112.18 |  | TRIDC2BG017100 | 122.49 |
|  | S2B_42277651 | 2B | 42.28 | TraesCS2B01G077200 | 42.28 |  | TraesCS2B03G0173000 | 48.36 |  | TRIDC2BG008340 | 49.48 |
|  | S2B_754153049 | 2B | 754.15 | TraesCS2B01G561600 | 754.15 |  | TraesCS2B03G1406500 | 762.45 |  | TRIDC2BG081160 | 772.29 |
|  | S3A_38433125 | 3A | 38.43 | TraesCS3A01G065200 | 38.44 |  | TraesCS3A03G0139300 | 39.32 |  | TRIDC3AG007600 | 38.94 |
|  | S3A_675295616 | 3A | 675.3 | TraesCS3A01G577600LC | 675.30 |  | TraesCS3A03G1008000LC | 675.46 |  | TRIDC3AG061360 | 675.47 |
|  | S3B_55404315 | 3B | 55.4 | TraesCS3B01G087300 | 55.40 |  | TraesCS3B03G0202200 | 66.31 |  | TRIDC3BG011890 | 68.01 |
|  | S4B_593136973 | 4B | 593.14 | TraesCS4B01G304800 | 593.14 |  | TraesCS4B03G0797300 | 592.13 |  | TRIDC4BG051930 | 604.44 |
|  | S4B_615781324 | 4B | 615.78 | TraesCS4B01G324900 | 615.77 |  | TraesCS4B03G0842500 | 614.61 |  | TRIDC4BG054630 | 627.16 |
|  | S5A_495771738 | 5A | 495.77 | TraesCS5A01G288000 | 495.82 |  | TraesCS5A03G0705400 | 496.36 |  | TRIDC5AG043150 | 444.62 |
|  | S7A_26878648 | 7A | 26.88 | TraesCS7A01G056000 | 26.88 |  | TraesCS7A03G0125500 | 27.82 |  | TRIDC7AG005840 | 25.72 |
|  | S7A_6298660 | 7A | 6.3 | TraesCS7A01G013700 | 6.30 |  | TraesCS7A03G0029500 | 6.63 |  | TRIDC0UG017450 | 4.41 |
|  | S7B_743147375 | 7B | 743.15 | TraesCS7B01G488900 | 743.14 |  | TraesCS7B03G1342500 | 763.84 |  | TRIDC7BG075060 | 777.55 |
| Abbreviations: SNP, single nucleotide polymorphism; Chr, chromosome; Mbp, mega base pairs. | | | | | | | | | | | |
