# Supplementary material for: Genome‐wide association studies on resistance to powdery mildew in cultivated emmer wheat
Source: Plant Genome. 2024 Jul 28;18(1):e20493. doi: 10.1002/tpg2.20493 (PMC11733656; doi:10.1002/tpg2.20493)
Supplement: Supplementary file 4 [file TPG2-18-e20493-s004.docx]

| **Supplemental Table S1.** Responses of 174 cultivated emmer wheat accessions to isolate *OKS(14)-B-3-1* of powdery mildew pathogen *Blumeria graminis* f. sp. *tritici* (*Bgt*). | | | | | | |
| --- | --- | --- | --- | --- | --- | --- |
| Panel no. | Accession^a^ | Country of origin or source | Infection type^b^ | | Resistance level | |
|  |  |  | Rep 1 | Rep 2 |  |  |
| 1 | CItr 7687-1 | Russian Federation | 0; | 0 |  | HR |
| 2 | CItr 12213-1 | India | 0 | 0 |  | HR |
| 3 | CItr 14133-1 | United States | 0 | 0 |  | HR |
| 4 | CItr 14621-1 | Ethiopia | 0; | 0; |  | HR |
| 5 | CItr 14637-1 | Ethiopia | 0 | 0 |  | HR |
| 6 | CItr 14916-1 | Unknown | 0; | 0; |  | HR |
| 7 | CItr 14917-1 | Ethiopia | 0 | 0; |  | HR |
| 8 | CItr 14919-1 | Unknown | 0 | 0 |  | HR |
| 9 | PI 41024-1 | Russian Federation | 0 | 0 |  | HR |
| 10 | PI 74108-1 | Georgia | 4 | 4 |  | HS |
| 11 | PI 94616-1 | Russian Federation | 3 | 3 |  | MS |
| 12 | PI 94621-1 | Armenia | 4 | 4 |  | HS |
| 13 | PI 94625-1 | Iran | 0; | 0; |  | HR |
| 14 | PI 94626-1 | Turkey | 3 | 3 |  | MS |
| 15 | PI 94627-1 | Asia Minor | 3 | 3 |  | MS |
| 16 | PI 94631-1 | Ethiopia | 0 | 0; |  | HR |
| 17 | PI 94634-1 | Morocco | 0 | 0 |  | HR |
| 18 | PI 94635-1 | Iran | 4 | 4 |  | HS |
| 19 | PI 94638-1 | Iran | 3 | 3 |  | MS |
| 20 | PI 94648-1 | Italy | 0; | 0 |  | HR |
| 21 | PI 94656-1 | Serbia | 0 | 0 |  | HR |
| 22 | PI 94664-1 | Saudi Arabia | 0; | 0; |  | HR |
| 23 | PI 94666-1 | Russian Federation | 4 | 4 |  | HS |
| 24 | PI 94673-1 | Armenia | 4 | 4 |  | HS |
| 25 | PI 94674-1 | Georgia | 4 | 4 |  | HS |
| 26 | PI 94675-1 | Georgia | 3 | 3 |  | MS |
| 27 | PI 94676-1 | Russian Federation | 0 | 0; |  | HR |
| 28 | PI 94738-1 | Ukraine | 4 | 4 |  | HS |
| 29 | PI 94747-1 | Georgia | 0 | 0 |  | HR |
| 30 | PI 101971-1 | India | 0 | 0 |  | HR |
| 31 | PI 133134-1 | Peru | 0 | 0 |  | HR |
| 32 | PI 154582-1 | Taiwan | 0 | 0; |  | HR |
| 33 | PI 164578-1 | India | 0 | 0 |  | HR |
| 34 | PI 168673-1 | United States | 0 | 0 |  | HR |
| 35 | PI 193641-1 | Ethiopia | 0; | 0; |  | HR |
| 36 | PI 193873-1 | Ethiopia | 0 | 0; |  | HR |
| 37 | PI 193879-1 | Ethiopia | 0 | 0 |  | HR |
| 38 | PI 193880-1 | Ethiopia | 0 | 0; |  | HR |
| 39 | PI 193882-1 | Ethiopia | 0 | 0; |  | HR |
| 40 | PI 193883-1 | Ethiopia | 0; | 0; |  | HR |
| 41 | PI 194042-1 | Ethiopia | 0 | 0; |  | HR |
| 42 | PI 194375-1 | Ethiopia | 0 | 0; |  | HR |
| 43 | PI 197483-1 | Ethiopia | 0 | 0; |  | HR |
| 44 | PI 197485-1 | Ethiopia | 0; | 0; |  | HR |
| 45 | PI 217637-1 | India | 0 | 0 |  | HR |
| 46 | PI 217639-1 | India | 0 | 0 |  | HR |
| 47 | PI 217640-1 | India | 0 | 0 |  | HR |
| 48 | PI 221400-1 | Serbia | 0 | 0 |  | HR |
| 49 | PI 225332-1 | Iran | 4 | 4 |  | HS |
| 50 | PI 244341-1 | Ethiopia | 0 | 0 |  | HR |
| 51 | PI 254165-1 | Iran | 0; | 0; |  | HR |
| 52 | PI 254167-1 | Iran | 3 | 3 |  | MS |
| 53 | PI 254189-1 | Georgia | 1 | 1 |  | HR |
| 54 | PI 254190-1 | Russian Federation | [4](mailto:10@4) | 4 |  | HR |
| 55 | PI 272533-1 | Hungary | 0 | 0 |  | HR |
| 56 | PI 273981-1 | Ethiopia | 0 | 0 |  | HR |
| 57 | PI 275996-1 | Spain | 0 | 0 |  | HR |
| 58 | PI 298582-1 | Ethiopia | 0 | 0 |  | HR |
| 59 | PI 310471-1 | India | 0 | 0 |  | HR |
| 60 | PI 319869-1 | Turkey | 0 | 0; |  | HR |
| 61 | PI 322232-1 | India | 0 | 0 |  | HR |
| 62 | PI 324076-1 | India | 0 | 0 |  | HR |
| 63 | PI 349043-1 | Georgia | 4 | 4 |  | HS |
| 64 | PI 349046-1 | Georgia | 4 | 4 |  | HS |
| 65 | PI 352548-1 | Ethiopia | 3 | 3 |  | MS |
| 66 | PI 355477-1 | Canada | 0 | 0 |  | HR |
| 67 | PI 355507-1 | Northwest Turkey | 4 | 4 |  | HS |
| 68 | PI 377655-1 | Former Yugoslavia | 0 | 0 |  | HR |
| 69 | PI 377657-1 | Former Yugoslavia | 0 | 0; |  | HR |
| 70 | PI 384332-1 | Ethiopia | 0; | [0;](mailto:10@0;) |  | HR |
| 71 | PI 434992-1 | Montenegro | 1 | [1](mailto:10@1) |  | HR |
| 72 | PI 480460-1 | Ethiopia | 4 | 4 |  | HS |
| 73 | PI 532305-1 | Oman | 2 | 2 |  | MR |
| 74 | CItr 3686 | United States | 1 | 1 |  | HR |
| 75 | CItr 4013 | India | 0 | 0 |  | HR |
| 76 | CItr 7685 | Russian Federation | 4 | 4 |  | HS |
| 77 | CItr 7686 | Russian Federation | 4 | 4 |  | HS |
| 78 | CItr 7779 | Ethiopia | 1 | 1 |  | HR |
| 79 | CItr 7962 | Ethiopia | 0 | 0; |  | HR |
| 80 | CItr 14085 | Unknown | 0 | 0 |  | HR |
| 81 | CItr 14086 | Unknown | 0 | 0 |  | HR |
| 82 | CItr 14098 | Ethiopia | 0 | 0 |  | HR |
| 83 | CItr 14639 | Ethiopia | 0; | 0; |  | HR |
| 84 | CItr 14751 | Ethiopia | 0 | 0; |  | HR |
| 85^c^ | CItr 14822 | Unknown | 4 | 0;/4 |  | seg |
| 86 | CItr 14834 | Ethiopia | 0 | 0; |  | HR |
| 87 | CItr 14866 | Ethiopia | 0 | 0 |  | HR |
| 88 | CItr 14971 | Unknown | 4 | 4 |  | HS |
| 89 | PI 41025 | Russian Federation | 0 | 0 |  | HR |
| 90 | PI 58788 | Ethiopia | 0 | 0 |  | HR |
| 91 | PI 60704 | Ethiopia | 0 | 0 |  | HR |
| 92 | PI 74106 | Iran | 4 | 4 |  | HS |
| 93 | PI 94617 | Russian Federation | 3 | 3 |  | MS |
| 94 | PI 94630 | Ethiopia | 0 | 0 |  | HR |
| 95 | PI 94654 | Bulgaria | 0 | 0 |  | HR |
| 96 | PI 94663 | Germany | 4 | 4 |  | HS |
| 97 | PI 94665 | Ethiopia | 0 | 0 |  | HR |
| 98 | PI 94668 | Russian Federation | 3 | 3 |  | MS |
| 99 | PI 94680 | Germany | 1 | 1 |  | HR |
| 100^c^ | PI 113961 | Georgia | 4/0 | 0;/4 |  | seg |
| 101 | PI 168675 | Ethiopia | 0 | 0 |  | HR |
| 102 | PI 190920 | Portugal | 4 | 4 |  | HS |
| 103 | PI 190926 | Belgium | 1 | 1 |  | HR |
| 104 | PI 191091 | Spain | 4 | 4 |  | HS |
| 105 | PI 193643 | Ethiopia | 0; | 0; |  | HR |
| 106 | PI 193878 | Ethiopia | 0 | 0 |  | HR |
| 107 | PI 195721 | Ethiopia | 0 | 0 |  | HR |
| 108 | PI 196100 | Ethiopia | 0; | 0; |  | HR |
| 109 | PI 196905 | Ethiopia | 0; | 0; |  | HR |
| 110 | PI 197482 | Ethiopia | 0 | 0 |  | HR |
| 111 | PI 197490 | Ethiopia | 0; | 0 |  | HR |
| 112 | PI 221401 | Serbia | 0 | 0 |  | HR |
| 113 | PI 226951 | Ethiopia | 0; | 0 |  | HR |
| 114 | PI 248991 | India | 0 | 0 |  | HR |
| 115 | PI 254146 | Ethiopia | 0; | 0; |  | HR |
| 116 | PI 254163 | Iran | 1 | 1 |  | HR |
| 117 | PI 254188 | Former Soviet Union | 4 | 4 |  | HS |
| 118 | PI 254193 | Spain | 1 | 1 |  | HR |
| 121 | PI 273982 | Ethiopia | 0; | 0 |  | HR |
| 123 | PI 275998 | Spain | 4 | 4 |  | HS |
| 124 | PI 275999 | Spain | 4 | 4 |  | HS |
| 125 | PI 276000 | Spain | 4 | 4 |  | HS |
| 126 | PI 276005 | Spain | - | 4 |  | HS |
| 127 | PI 276006 | Spain | 4 | 4 |  | HS |
| 128 | PI 276007 | Spain | 4 | 4 |  | HS |
| 129 | PI 276012 | Spain | 4 | 4 |  | HS |
| 130 | PI 276014 | Spain | 4 | 4 |  | HS |
| 131 | PI 277670 | Spain | 1 | 1 |  | HR |
| 134 | PI 286061 | Poland | 1 | 1 |  | HR |
| 135 | PI 289603 | United Kingdom | 1 | 1 |  | HR |
| 136 | PI 295065 | Bulgaria | 0 | 0 |  | HR |
| 137 | PI 297830 | Ethiopia | 0 | 0; |  | HR |
| 138 | PI 298543 | Ethiopia | 0 | 0 |  | HR |
| 139 | PI 298548 | Ethiopia | 0; | - |  | HR |
| 140 | PI 306536 | Romania | 2 | 2 |  | MR |
| 141 | PI 330544 | United Kingdom | 4 | 4 |  | HS |
| 142 | PI 349045 | Russian Federation | 4 | 4 |  | HS |
| 143 | PI 350001 | Serbia | 0 | 0 |  | HR |
| 144 | PI 352335 | United States | 1 | 1 |  | HR |
| 145 | PI 352337 | Spain | 4 | 4 |  | HS |
| 146 | PI 352338 | Spain | 4 | 4 |  | HS |
| 147 | PI 352341 | Spain | 4 | 4 |  | HS |
| 148 | PI 352342 | Spain | 4 | 4 |  | HS |
| 149 | PI 352358 | France | 1 | 1 |  | HR |
| 150 | PI 352365 | Germany | 1 | 1 |  | HR |
| 151 | PI 355460 | Switzerland | 1 | 1 |  | HR |
| 153 | PI 355470 | Germany | 2 | 2 |  | MR |
| 154 | PI 355475 | Germany | 4 | 4 |  | HS |
| 156 | PI 355485 | Spain | - | 4 |  | HS |
| 157 | PI 355486 | Spain | - | 4 |  | HS |
| 158 | PI 355489 | France | 4 | 4 |  | HS |
| 159 | PI 355497 | Former Soviet Union | 4 | 4 |  | HS |
| 160 | PI 355505 | Ancient Palestine | 4 | 4 |  | HS |
| 161 | PI 361833 | Denmark | 4 | 4 |  | HS |
| 162 | PI 362438 | Serbia | 0 | 0 |  | HR |
| 163 | PI 362500 | Serbia | 2 | 2 |  | MR |
| 164 | PI 362697 | Montenegro | 0 | 0 |  | HR |
| 165 | PI 374685 | Bosnia and Herzegovina | 0; | 0 |  | HR |
| 166 | PI 377650 | Former Yugoslavia | 0; | 0; |  | HR |
| 167 | PI 377672 | Former Yugoslavia | 0 | 0 |  | HR |
| 168 | PI 384297 | Ethiopia | 0; | 0; |  | HR |
| 169 | PI 384302 | Ethiopia | 0; | 0; |  | HR |
| 170 | PI 384318 | Ethiopia | 1 | 1 |  | HR |
| 171 | PI 384331 | Ethiopia | 0; | 0; |  | HR |
| 172 | PI 434996 | Montenegro | 0 | 0 |  | HR |
| 173 | PI 470737 | Northeast Turkey | 4 | 4 |  | HS |
| 174 | PI 479957 | Ethiopia | 3 | 3 |  | MS |
| 175 | PI 479965 | Ethiopia | 0; | 0 |  | HR |
| 176 | PI 480307 | Ethiopia | 0; | 0; |  | HR |
| 177 | PI 480312 | Ethiopia | 0 | 0; |  | HR |
| 178 | PI 480313 | Ethiopia | 0; | 0; |  | HR |
| 179 | PI 480461 | Ethiopia | 0; | 0; |  | HR |
| 180 | PI 480462 | Ethiopia | 0; | 0; |  | HR |
| 181 | PI 532304 | Oman | 2 | 2 |  | MR |
| 182 | KL-B | United States | 0 | 0 |  | HR |
| 192 | Rusty | United States | 4 | 4 |  | HS |
| ^a^Accession numbers and sources or origins of the accessions were obtained from USDA-ARS National Plant Germplasm System (https://npgsweb.ars-grin.gov/gringlobal/search) (Aberdeen, Idaho, USA). The accession numbers followed by “-1” (e.g. CItr 14916-1) indicates a single plant selection. | | | | | | |
| ^b^IT score of “0”, “0;” and “1” are considered highly resistant (HR), “2” as moderately resistant (MR), “3” as moderately susceptible (MS), “4” as highly susceptible (HS), segregating (seg) and “-” for unavailable data. | | | | | | |
| ^c^Seeds of panel no. 85 and 100 were segregating, hence, some variation was observed within a replicate or between replicates. | | | | | | |
