# Supplementary material for: Genome‐wide association studies on resistance to powdery mildew in cultivated emmer wheat
Source: Plant Genome. 2024 Jul 28;18(1):e20493. doi: 10.1002/tpg2.20493 (PMC11733656; doi:10.1002/tpg2.20493)
Supplement: Supplementary file 2 — Supplemental Table S2. Normality and homogeneity tests of the replicated samples in response to B. graminis f. sp. tritici (Bgt) isolate OKS(14)‐B‐3‐1. [file TPG2-18-e20493-s003.docx]

| **Supplemental Table S2.** Normality and homogeneity tests of the replicated samples in response to *B. graminis* f. sp. *tritici* (*Bgt*) isolate *OKS(14)-B-3-1.* | | | | |
| --- | --- | --- | --- | --- |
| Shapiro-Wilk normality test | |  | Fligner-Killeen test of homogeneity (Rep 1 vs. Rep 2) | |
| W | 0.68 |  | Chi-squared | 3.41 |
| *p*-value | 2.20E-16 |  | df | 4.00 |
|  |  |  | *p*-value | 0.49 |
