# Supplementary material for: Genome‐wide association studies on resistance to powdery mildew in cultivated emmer wheat
Source: Plant Genome. 2024 Jul 28;18(1):e20493. doi: 10.1002/tpg2.20493 (PMC11733656; doi:10.1002/tpg2.20493)
Supplement: Supplementary file 5 — Supplemental Table S5. Alternative alleles of significant SNPs derived from GBS for all cultivated emmer accessions. [file TPG2-18-e20493-s001.docx]

| **Supplemental Table S5.** Alternative alleles of significant SNPs derived from GBS for all cultivated emmer accessions. | | | | | | | | | | | | | | | | | | | | | | |
| --- | --- | --- | --- | --- | --- | --- | --- | --- | --- | --- | --- | --- | --- | --- | --- | --- | --- | --- | --- | --- | --- | --- |
| Accession | S1B_18564537 | S1B_561151612 | S2A_38557560 | S2A_78559456 | S2A_105044730 | S2A_775441196 | S2B_42277651 | S2B_104577357 | S2B_754153049 | S3A_38433125 | S3A_675295616 | S3B_55404315 | S4B_593136973 | S4B_615781324 | S5A_495771738 | S7A_6298660 | S7A_26878648 | S7B_743147375 | Source or origin | | Mean infection type | Resistance level |
| PI 374685 | A | A | C | G | C | G | A | T | C | G | A | T | T | A | A | G | T | C | Bosnia & Herzegovina | | 0.0 | HR |
| PI 94654 | G | C | T | C | T | A | A | C | A | G | A | T | - | C | A | - | T | T | Bulgaria | | 0.0 | HR |
| PI 295065 | G | C | C | G | C | G | A | T | A | G | A | T | T | A | A | C | G | C | Bulgaria | | 0.0 | HR |
| PI 355477-1 | G | C | C | G | C | G | T | T | C | G | A | T | T | A | A | C | T | C | Canada | | 0.0 | HR |
| CItr 14098 | A | A | C | C | T | G | T | T | A | G | A | C | T | A | A | G | G | C | Ethiopia | | 0.0 | HR |
| CItr 14621-1 | A | A | C | C | T | G | T | T | A | G | A | C | T | A | A | G | G | C | Ethiopia | | 0.0 | HR |
| CItr 14637-1 | A | A | C | C | T | G | T | T | A | G | A | C | T | A | A | G | G | C | Ethiopia | | 0.0 | HR |
| CItr 14639 | A | A | C | C | T | G | T | T | A | G | A | C | T | A | A | G | G | C | Ethiopia | | 0.0 | HR |
| CItr 14751 | A | A | C | C | T | G | T | T | A | G | A | C | T | A | A | G | G | C | Ethiopia | | 0.0 | HR |
| CItr 14834 | A | A | C | C | T | G | T | T | A | G | A | C | T | A | A | G | G | C | Ethiopia | | 0.0 | HR |
| CItr 14866 | A | A | C | C | T | G | T | T | A | G | A | C | T | A | A | G | G | C | Ethiopia | 0.0 | | HR |
| CItr 14917-1 | A | A | C | C | T | G | T | T | A | G | A | C | T | A | A | G | G | C | Ethiopia | 0.0 | | HR |
| CItr 7962 | A | A | C | C | T | G | T | T | A | G | A | C | T | A | A | G | G | C | Ethiopia | 0.0 | | HR |
| PI 168675 | A | A | C | C | T | G | T | T | A | G | A | C | T | A | A | G | G | C | Ethiopia | 0.0 | | HR |
| PI 193641-1 | A | A | C | C | T | G | T | T | A | G | A | C | T | A | A | G | G | C | Ethiopia | 0.0 | | HR |
| PI 193873-1 | A | A | C | C | T | G | T | T | A | G | A | C | T | A | A | G | G | C | Ethiopia | 0.0 | | HR |
| PI 193878 | A | A | C | C | T | G | T | T | A | G | A | C | T | A | A | G | G | C | Ethiopia | 0.0 | | HR |
| PI 193879-1 | A | A | C | C | T | G | T | T | A | G | A | C | T | A | A | G | G | C | Ethiopia | 0.0 | | HR |
| PI 193880-1 | A | A | C | C | T | G | T | T | A | G | A | C | T | A | A | G | G | C | Ethiopia | 0.0 | | HR |
| PI 193882-1 | A | A | C | C | T | G | T | T | A | G | A | C | T | A | A | G | G | C | Ethiopia | 0.0 | | HR |
| PI 193883-1 | A | A | C | C | T | G | T | T | A | G | A | C | T | A | A | G | G | C | Ethiopia | 0.0 | | HR |
| PI 194042-1 | A | A | C | C | T | G | T | T | A | G | A | C | T | A | A | G | G | C | Ethiopia | 0.0 | | HR |
| PI 194375-1 | A | A | C | C | T | G | T | T | A | G | A | C | T | A | A | G | G | C | Ethiopia | 0.0 | | HR |
| PI 195721 | A | A | C | C | T | - | T | T | A | G | - | - | T | A | A | G | G | C | Ethiopia | 0.0 | | HR |
| PI 196100 | A | A | C | C | T | G | T | T | A | G | A | C | T | A | A | G | G | C | Ethiopia | 0.0 | | HR |
| PI 197482 | A | A | C | C | T | G | T | T | A | G | A | C | T | A | A | G | G | C | Ethiopia | 0.0 | | HR |
| PI 197483-1 | A | A | C | C | T | G | T | T | A | G | A | C | T | A | A | G | G | C | Ethiopia | 0.0 | | HR |
| PI 197485-1 | A | A | C | C | T | G | T | T | A | G | A | C | T | A | A | G | G | C | Ethiopia | 0.0 | | HR |
| PI 197490 | A | A | C | C | T | G | T | T | A | G | A | C | T | A | A | G | G | C | Ethiopia | 0.0 | | HR |
| PI 58788 | A | A | C | C | T | G | T | T | A | G | A | C | T | A | A | G | G | C | Ethiopia | 0.0 | | HR |
| PI 60704 | A | A | C | C | T | G | T | T | A | G | A | C | T | A | A | G | G | C | Ethiopia | 0.0 | | HR |
| PI 94630 | A | A | C | C | T | G | T | T | A | G | A | C | T | A | A | G | G | C | Ethiopia | 0.0 | | HR |
| PI 94631-1 | A | A | C | C | T | G | T | T | A | G | A | C | T | A | A | G | G | C | Ethiopia | 0.0 | | HR |
| PI 244341-1 | A | A | C | C | T | G | T | T | A | G | A | C | T | A | A | G | G | C | Ethiopia | 0.0 | | HR |
| PI 254146 | A | A | C | C | T | G | T | T | A | G | A | C | T | A | A | G | G | C | Ethiopia | 0.0 | | HR |
| PI 273981-1 | A | A | C | C | T | G | T | T | A | G | A | C | T | A | A | G | G | C | Ethiopia | 0.0 | | HR |
| PI 273982 | A | A | C | C | T | G | T | T | A | G | A | C | T | A | A | G | G | C | Ethiopia | 0.0 | | HR |
| PI 297830 | A | A | C | C | T | G | T | T | A | G | A | C | T | A | A | G | G | C | Ethiopia | 0.0 | | HR |
| PI 298543 | A | A | C | C | T | G | T | T | A | G | A | C | T | A | A | G | G | C | Ethiopia | 0.0 | | HR |
| PI 298548 | A | A | C | C | T | G | T | T | A | G | A | C | T | A | A | G | G | C | Ethiopia | 0.0 | | HR |
| PI 298582-1 | A | A | C | C | T | G | T | T | A | G | A | C | T | A | A | G | G | C | Ethiopia | 0.0 | | HR |
| PI 384297 | A | A | C | C | T | G | T | T | A | G | A | C | T | A | A | G | G | C | Ethiopia | 0.0 | | HR |
| PI 384302 | A | A | C | C | T | G | T | T | A | G | A | C | T | A | A | G | G | C | Ethiopia | 0.0 | | HR |
| PI 384331 | A | A | C | C | T | G | T | T | A | G | A | C | T | A | A | G | G | C | Ethiopia | 0.0 | | HR |
| PI 384332-1 | A | A | C | C | T | G | T | T | A | G | A | C | T | A | A | G | G | C | Ethiopia | 0.0 | | HR |
| PI 479965 | A | A | C | C | T | G | T | T | A | G | A | C | T | A | A | G | G | C | Ethiopia | 0.0 | | HR |
| PI 480307 | A | A | C | C | T | G | T | T | A | G | A | C | T | A | A | G | G | C | Ethiopia | 0.0 | | HR |
| PI 480312 | A | A | C | C | T | G | T | T | A | G | A | C | T | A | A | G | G | C | Ethiopia | 0.0 | | HR |
| PI 480313 | A | A | C | C | T | G | T | T | A | G | A | C | T | A | A | G | G | C | Ethiopia | 0.0 | | HR |
| PI 480461 | A | A | C | C | T | G | T | T | A | G | A | C | T | A | A | G | G | C | Ethiopia | 0.0 | | HR |
| PI 480462 | A | A | C | C | T | G | T | T | A | G | A | C | T | A | A | G | G | C | Ethiopia | 0.0 | | HR |
| PI 196905 | A | A | C | C | T | G | A | T | A | G | A | C | T | A | A | C | G | C | Ethiopia | 0.0 | | HR |
| PI 226951 | G | A | T | C | T | G | A | T | A | G | A | T | T | A | A | G | G | C | Ethiopia | 0.0 | | HR |
| PI 94665 | G | C | C | C | T | G | A | T | A | G | A | T | - | C | A | G | T | T | Ethiopia | 0.0 | | HR |
| CItr 12213-1 | A | A | C | C | - | A | T | T | A | G | A | C | T | A | A | C | T | C | India | 0.0 | | HR |
| PI 101971-1 | A | A | C | C | T | A | T | T | A | G | A | C | T | A | A | C | T | C | India | 0.0 | | HR |
| PI 164578-1 | A | A | C | C | T | A | T | T | A | G | A | C | T | A | A | C | T | C | India | 0.0 | | HR |
| PI 217637-1 | A | A | C | C | T | A | T | T | A | G | A | C | T | A | A | C | T | C | India | 0.0 | | HR |
| PI 217639-1 | A | A | C | C | T | A | T | T | A | G | A | C | T | A | A | C | T | C | India | 0.0 | | HR |
| PI 248991 | A | A | C | C | T | A | T | T | A | G | A | C | T | A | A | C | T | C | India | 0.0 | | HR |
| PI 310471-1 | A | A | C | C | T | A | T | T | A | G | A | C | T | A | A | C | T | C | India | 0.0 | | HR |
| PI 322232-1 | A | A | C | C | T | A | T | T | A | G | A | C | T | A | A | C | T | C | India | 0.0 | | HR |
| PI 324076-1 | A | A | C | C | T | A | T | T | A | G | A | C | T | A | A | C | T | C | India | 0.0 | | HR |
| PI 217640-1 | A | A | C | C | T | G | T | T | A | G | A | C | T | A | A | G | G | C | India | 0.0 | | HR |
| CItr 4013 | A | A | C | G | C | G | A | T | C | G | A | T | T | C | A | C | T | C | India | 0.0 | | HR |
| PI 275996-1 | A | A | C | C | T | A | T | T | A | G | A | C | T | A | A | C | T | C | Spain | 0.0 | | HR |
| PI 94747-1 | A | A | C | C | T | G | T | T | A | G | A | C | T | A | A | G | T | C | Georgia | 0.0 | | HR |
| PI 94664-1 | A | A | C | C | T | G | T | T | A | G | A | C | T | A | A | G | G | C | Saudi Arabia | 0.0 | | HR |
| PI 94656-1 | A | A | C | C | T | A | T | T | A | G | A | C | T | A | A | C | T | C | Serbia | 0.0 | | HR |
| PI 319869-1 | A | A | C | C | T | G | T | T | A | G | A | C | T | A | A | G | G | C | Turkey | 0.0 | | HR |
| PI 168673-1 | A | A | C | C | T | G | T | T | A | G | A | C | T | A | A | G | G | C | United States | 0.0 | | HR |
| PI 94634-1 | - | A | T | C | T | A | T | T | A | G | A | T | T | A | A | C | G | C | Morocco | 0.0 | | HR |
| PI 133134-1 | A | A | C | C | T | A | T | T | A | G | A | C | T | A | A | C | T | C | Peru | 0.0 | | HR |
| PI 41024-1 | A | A | C | C | T | A | T | T | A | G | A | C | T | A | A | C | T | C | Russian Federation | 0.0 | | HR |
| PI 94676-1 | A | A | C | C | T | G | T | T | A | G | A | C | T | A | A | C | T | C | Russian Federation | 0.0 | | HR |
| PI 41025 | A | A | C | G | C | G | A | T | C | G | A | T | T | C | A | G | T | C | Russian Federation | 0.0 | | HR |
| CItr 7687-1 | G | C | T | C | T | A | A | C | A | G | A | T | - | C | A | G | T | T | Russian Federation | 0.0 | | HR |
| PI 221400-1 | A | A | C | G | C | A | A | T | C | G | A | T | T | A | A | C | T | C | Serbia | 0.0 | | HR |
| PI 221401 | A | A | C | G | C | A | A | T | C | G | A | T | T | A | A | C | T | C | Serbia | 0.0 | | HR |
| PI 350001 | A | A | C | G | C | A | A | T | C | G | A | T | T | A | A | C | T | C | Serbia | 0.0 | | HR |
| PI 362438 | A | A | C | G | C | A | A | T | C | G | A | T | T | A | A | G | T | C | Serbia | 0.0 | | HR |
| PI 377655-1 | A | A | C | G | C | A | A | T | C | G | A | T | T | C | A | C | T | C | Former Yugoslavia | 0.0 | | HR |
| PI 377650 | A | A | C | G | C | G | A | T | C | G | A | T | T | C | A | G | T | C | Former Yugoslavia | 0.0 | | HR |
| PI 377657-1 | A | A | C | G | C | G | A | T | C | G | A | T | T | C | A | G | T | C | Former Yugoslavia | 0.0 | | HR |
| PI 377672 | A | A | C | G | C | G | A | T | C | G | A | T | T | A | A | C | T | C | Former Yugoslavia | 0.0 | | HR |
| PI 94648-1 | A | A | C | G | C | A | A | T | C | G | A | T | T | C | A | C | T | C | Italy | 0.0 | | HR |
| PI 434996 | A | A | C | G | C | A | A | T | C | G | A | T | C | C | A | C | T | C | Montenegro | 0.0 | | HR |
| PI 362697 | A | A | C | G | C | G | A | T | C | G | A | T | T | A | A | G | T | C | Montenegro | 0.0 | | HR |
| CItr 14919-1 | A | A | C | C | T | A | T | T | A | G | A | C | T | A | A | C | T | C | Unknown | 0.0 | | HR |
| CItr 14916-1 | A | A | C | C | T | G | T | T | A | G | A | C | T | A | A | G | G | C | Unknown | 0.0 | | HR |
| CItr 14085 | G | A | C | G | C | A | T | T | C | A | A | T | T | A | A | C | G | C | Unknown | 0.0 | | HR |
| CItr 14086 | G | C | C | G | C | A | T | T | C | A | A | T | T | A | A | C | G | C | Unknown | 0.0 | | HR |
| PI 94625-1 | G | C | T | C | T | A | A | C | A | G | A | T | - | C | A | G | T | T | Iran | 0.0 | | HR |
| PI 254165-1 | G | C | T | C | C | A | A | C | A | G | A | T | T | A | A | G | T | C | Iran | 0.0 | | HR |
| PI 272533-1 | G | C | T | C | C | A | A | T | A | G | A | T | T | C | A | G | T | T | Hungary | 0.0 | | HR |
| PI 154582-1 | A | C | C | G | C | A | T | T | C | G | T | C | C | A | A | C | G | C | Taiwan | 0.0 | | HR |
| PI 193643 | A | A | C | C | T | G | T | T | A | G | A | C | T | A | A | G | G | C | Ethiopia | 0.5 | | HR |
| PI 384318 | A | A | C | C | T | G | T | T | A | G | A | C | T | A | A | G | G | C | Ethiopia | 1.0 | | HR |
| PI 434992-1 | A | A | C | G | C | A | A | T | C | G | A | T | C | C | A | C | T | C | Montenegro | 1.0 | | HR |
| PI 277670 | A | C | C | C | T | G | T | T | C | G | T | T | C | C | G | C | T | C | Spain | 1.0 | | HR |
| PI 254193 | G | C | C | G | C | G | T | T | C | A | A | T | T | A | A | C | T | C | Spain | 1.0 | | HR |
| PI 190926 | G | C | C | G | C | G | T | T | C | A | A | T | T | A | A | C | T | C | Belgium | 1.0 | | HR |
| PI 352358 | G | C | C | G | C | G | T | T | C | A | A | T | T | A | A | C | T | C | France | 1.0 | | HR |
| PI 94680 | G | C | C | G | C | G | T | T | C | A | A | T | T | A | A | C | T | C | Germany | 1.0 | | HR |
| PI 352365 | G | C | C | G | C | G | T | T | C | A | A | T | T | A | A | C | T | C | Germany | 1.0 | | HR |
| PI 286061 | G | C | C | G | C | G | T | T | C | A | A | T | T | A | A | C | T | C | Poland | 1.0 | | HR |
| PI 355460 | G | C | C | G | C | G | T | T | C | A | A | T | T | A | A | C | T | C | Switzerland | 1.0 | | HR |
| PI 289603 | G | C | C | G | C | G | T | T | C | A | A | T | T | A | A | C | T | C | United Kingdom | 1.0 | | HR |
| PI 254189-1 | G | C | T | C | T | A | A | T | A | G | A | T | - | A | G | G | T | T | Georgia | 1.0 | | HR |
| PI 254163 | G | C | T | C | C | A | A | C | A | G | A | T | T | A | A | G | G | C | Iran | 1.0 | | HR |
| CItr 3686 | G | C | T | C | C | A | A | C | A | G | A | T | T | C | A | G | T | T | United States | 1.0 | | HR |
| PI 352335 | G | C | T | C | C | A | A | C | A | G | A | T | T | C | A | G | T | T | United States | 1.0 | | HR |
| PI 532304 | A | A | C | C | T | A | T | T | A | G | A | T | T | A | A | G | T | C | Oman | 2.0 | | MR |
| PI 532305-1 | A | A | C | C | T | A | T | T | A | G | A | T | T | A | A | G | T | C | Oman | 2.0 | | MR |
| PI 355470 | A | A | C | C | T | G | A | T | A | G | A | T | T | A | G | C | G | C | Germany | 2.0 | | MR |
| PI 362500 | A | A | C | G | C | G | A | T | C | G | A | T | T | A | A | C | T | C | Serbia | 2.0 | | MR |
| PI 113961 | G | A | T | C | C | A | A | T | A | G | A | T | T | C | A | G | T | C | Georgia | 2.0 | | MR |
| PI 306536 | G | C | C | G | C | G | A | T | C | A | A | T | T | A | A | C | G | C | Romania | 2.0 | | MR |
| PI 479957 | A | A | C | C | T | G | T | T | A | G | A | T | T | A | A | G | G | C | Ethiopia | 3.0 | | MS |
| PI 352548-1 | A | A | C | C | C | G | A | T | A | G | A | T | T | C | G | G | T | C | Ethiopia | 3.0 | | MS |
| PI 94627-1 | A | A | C | C | C | G | A | T | C | G | A | T | C | C | A | G | T | C | Asia Minor | 3.0 | | MS |
| PI 94616-1 | A | A | C | C | C | G | A | T | C | G | A | T | C | C | A | G | T | C | Russian Federation | 3.0 | | MS |
| PI 94617 | A | A | C | C | C | G | A | T | C | G | A | T | T | C | A | G | T | C | Russian Federation | 3.0 | | MS |
| PI 94626-1 | - | A | C | G | C | G | A | T | C | G | A | T | C | C | A | C | T | T | Turkey | 3.0 | | MS |
| PI 94668 | G | C | C | C | T | G | A | C | A | G | A | T | T | C | G | G | T | T | Russian Federation | 3.0 | | MS |
| PI 94675-1 | G | C | T | C | T | G | A | C | - | G | A | T | T | C | G | G | T | T | Georgia | 3.0 | | MS |
| PI 94638-1 | G | C | C | C | C | G | T | T | A | G | A | T | T | A | G | C | T | C | Iran | 3.0 | | MS |
| PI 254167-1 | G | C | T | C | C | A | A | T | - | G | A | T | - | C | A | G | T | T | Iran | 3.0 | | MS |
| PI 361833 | A | A | C | C | T | G | T | T | A | G | A | T | T | A | A | G | G | C | Denmark | 4.0 | | HS |
| PI 480460-1 | A | A | C | C | T | G | T | T | A | G | A | T | T | A | A | G | G | C | Ethiopia | 4.0 | | HS |
| CItr 14822 | A | A | C | C | T | G | A | T | A | G | A | C | T | A | A | C | G | C | Unknown | 4.0 | | HS |
| CItr 14971 | A | A | C | C | C | G | A | T | C | G | A | T | C | A | A | C | T | C | Unknown | 4.0 | | HS |
| PI 355507-1 | A | A | C | C | C | G | A | T | C | G | A | T | C | C | A | G | T | C | Northwest Turkey | 4.0 | | HS |
| PI 254188 | A | A | C | C | C | G | A | T | C | G | A | T | C | C | A | C | T | C | Former Soviet Union | 4.0 | | HS |
| PI 355497 | A | C | C | C | C | A | A | T | - | G | A | T | C | A | G | C | T | C | Former Soviet Union | 4.0 | | HS |
| PI 355505 | A | C | C | G | C | G | T | T | C | A | A | T | C | A | A | C | G | C | Ancient Palestine | 4.0 | | HS |
| PI 94663 | - | C | T | C | T | A | A | C | C | A | A | T | T | A | A | C | G | T | Germany | 4.0 | | HS |
| PI 355475 | A | C | C | C | T | G | T | T | C | A | A | T | C | A | A | C | G | C | Germany | 4.0 | | HS |
| PI 190920 | A | C | C | C | T | G | T | T | C | A | A | T | C | A | G | C | G | C | Portugal | 4.0 | | HS |
| PI 191091 | - | C | C | C | T | G | T | T | C | A | T | T | C | C | G | C | T | C | Spain | 4.0 | | HS |
| PI 275998 | A | C | C | C | T | G | T | T | C | A | A | T | C | C | G | C | T | C | Spain | 4.0 | | HS |
| PI 275999 | A | C | C | C | T | G | T | T | C | A | A | T | C | C | G | C | T | C | Spain | 4.0 | | HS |
| PI 276000 | A | C | C | C | T | G | T | T | C | A | A | T | C | C | G | - | T | C | Spain | 4.0 | | HS |
| PI 276005 | A | A | C | C | T | G | T | T | C | A | A | T | C | C | G | C | T | C | Spain | 4.0 | | HS |
| PI 276006 | A | C | C | C | T | G | T | T | C | A | T | T | C | C | G | C | T | C | Spain | 4.0 | | HS |
| PI 276007 | A | C | C | C | T | G | T | T | C | G | A | T | C | C | G | C | T | C | Spain | 4.0 | | HS |
| PI 276012 | - | C | C | C | T | G | T | T | C | A | T | T | C | C | G | C | T | C | Spain | 4.0 | | HS |
| PI 276014 | A | C | C | C | T | G | T | T | C | A | T | T | C | C | G | C | T | C | Spain | 4.0 | | HS |
| PI 352337 | A | C | C | C | T | G | T | T | C | A | T | T | C | C | G | C | T | C | Spain | 4.0 | | HS |
| PI 352338 | A | C | C | C | T | G | T | T | C | A | T | T | C | C | G | C | T | C | Spain | 4.0 | | HS |
| PI 352341 | A | C | C | C | T | G | T | T | C | A | T | T | C | C | G | C | T | C | Spain | 4.0 | | HS |
| PI 352342 | A | C | C | C | T | G | T | T | C | A | T | T | C | C | G | C | T | C | Spain | 4.0 | | HS |
| PI 355485 | A | C | C | C | T | G | T | T | C | A | T | T | C | C | G | C | T | C | Spain | 4.0 | | HS |
| PI 355486 | A | C | C | C | T | G | T | T | C | A | T | T | C | C | G | C | G | C | Spain | 4.0 | | HS |
| PI 94738-1 | A | C | T | C | C | G | A | T | A | G | A | T | - | C | G | G | T | T | Ukraine | 4.0 | | HS |
| PI 330544 | A | C | C | C | T | G | T | T | C | A | A | T | C | C | G | C | T | C | United Kingdom | 4.0 | | HS |
| PI 74108-1 | A | C | T | C | C | G | A | T | A | G | A | T | - | A | A | C | T | T | Georgia | 4.0 | | HS |
| PI 94674-1 | A | C | T | C | C | A | A | T | A | G | A | T | - | C | G | C | T | T | Georgia | 4.0 | | HS |
| PI 349043-1 | G | C | T | C | C | G | A | C | A | G | A | T | - | C | G | G | T | T | Georgia | 4.0 | | HS |
| PI 349046-1 | G | C | T | C | C | A | A | T | - | G | A | T | - | C | G | C | T | T | Georgia | 4.0 | | HS |
| PI 94621-1 | G | C | C | C | C | G | A | C | A | G | A | T | T | C | G | G | T | T | Armenia | 4.0 | | HS |
| PI 94673-1 | G | C | C | C | C | G | A | C | A | G | A | T | T | C | G | G | T | T | Armenia | 4.0 | | HS |
| PI 355489 | G | C | C | G | C | G | T | T | C | G | A | T | T | A | G | C | T | C | France | 4.0 | | HS |
| CItr 7685 | A | C | C | C | T | G | T | T | C | A | T | T | C | C | G | C | T | C | Russian Federation | 4.0 | | HS |
| CItr 7686 | A | C | C | C | T | G | T | T | C | A | T | T | C | C | G | C | T | C | Russian Federation | 4.0 | | HS |
| PI 94666-1 | G | C | C | C | C | G | A | C | A | G | A | T | T | C | G | G | T | T | Russian Federation | 4.0 | | HS |
| PI 254190-1 | G | C | C | C | C | A | A | C | A | G | A | T | T | A | A | C | T | T | Russian Federation | 4.0 | | HR |
| PI 349045 | G | C | T | C | C | G | A | C | - | G | A | T | T | C | G | G | T | T | Russian Federation | 4.0 | | HS |
| PI 225332-1 | A | C | C | C | C | A | A | C | A | G | A | T | T | C | A | C | T | T | Iran | 4.0 | | HS |
| PI 74106 | G | C | T | C | C | G | A | C | A | G | A | T | T | C | A | C | T | T | Iran | 4.0 | | HS |
| PI 94635-1 | G | C | T | C | C | A | A | T | A | G | A | T | - | C | G | C | T | T | Iran | 4.0 | | HS |
| PI 470737 | G | C | C | C | C | G | A | T | A | G | A | T | - | A | G | G | T | T | Northeast Turkey | 4.0 | | HS |
| Resistant alleles are highlighted green and susceptible alleles are highlighted red. Infection type scores are colored from light yellow (0) to dark yellow (4). Missing alleles “-” are highlighted gray. | | | | | | | | | | | | | | | | | | | | | | |
| Highly resistant accessions with IT=0 for Ethiopian and Indian accessions and highly susceptible accession with IT=4 for Spanish accessions are boxed for comparisons. | | | | | | | | | | | | | | | | | | | | | | |
